# Prognostic value of prognostic nutritional index in breast cancer patients receiving neoadjuvant therapy: a systematic review and meta-analysis

**DOI:** 10.3389/fonc.2026.1775749

**Published:** 2026-04-27

**Authors:** Meihui Shan, Ziqian Zhao, Munawar Anwar, Jiawei Chen, Yuquan Dai, Yeliya Yeerboli, Zuqiang Xu, Zizhang Wang, Le Yang, Chao Dong

**Affiliations:** 1 The Clinical Medical Research Center of Breast and Thyroid Tumor in Xinjiang, Tumor Hospital Affiliated to Xinjiang Medical University, Urumqi, China; 2 Xinjiang Key Laboratory of Oncology, The Third Affiliated Teaching Hospital (Affiliated Cancer Hospital) of Xinjiang Medical University, Urumqi, China; 3 Department of Clinical Medicine, Xinjiang Medical University, Urumqi, China

**Keywords:** breast cancer, meta-analysis, neoadjuvant chemotherapy, prognosis, prognostic nutritional index

## Abstract

**Objective:**

Although the prognostic utility of the prognostic nutritional index (PNI) in breast cancer (BC) treated with neoadjuvant chemotherapy (NACT) has been widely reported, the available evidence remains inconsistent. Therefore, this meta-analysis systematically evaluated the predictive value of pre-NACT PNI for clinical outcomes in patients with BC.

**Methods:**

PubMed, Embase, Web of Science, and the Cochrane Library were queried comprehensively through November 2025 to identify reports evaluating the association of pre-NACT PNI with overall survival (OS), disease-free survival (DFS), and pathological complete response (pCR) in individuals with BC. Eligible studies were selected according to the PICOS framework. Hazard ratios (HRs) and odds ratios (ORs), along with 95% confidence intervals (CIs), were retrieved. Effect estimates were synthesized using fixed-or random-effects models as indicated, and robustness was tested by subgroup analyses, sensitivity analyses, and evaluation of publication bias.

**Results:**

Nine studies comprising 3,718 patients were included. A high pre-treatment PNI was significantly associated with better OS (HR = 0.36, 95% CI 0.25–0.52; p < 0.001) and increased odds of achieving pCR (OR = 1.90, 95% CI 1.21–2.98; p = 0.005). A favorable association was also observed for DFS (HR = 0.52, 95% CI 0.31–0.87; p = 0.01); however, this finding should be interpreted with caution because substantial heterogeneity was present and the estimate showed limited stability in leave-one-out sensitivity analyses. Subgroup analyses indicated that sample size, age, PNI cut-off value, and geographic region might modify the predictive performance of PNI for DFS and pCR.

**Conclusions:**

A high pre-NACT PNI was significantly associated with longer OS and a higher likelihood of pCR in patients with BC receiving NACT. A favorable association with DFS was also observed, but this finding should be interpreted cautiously because of substantial heterogeneity and limited robustness. PNI may serve as a simple and practical marker for clinical risk stratification, although further prospective validation is warranted.

**Systematic review registration:**

https://www.crd.york.ac.uk/prospero/, identifier CRD420251268856.

## 1 Introduction

The 2024 statistics published by the World Health Organization (1) indicate that breast cancer remains a major contributor to cancer-related morbidity and mortality. Worldwide, breast cancer incidence is reported to rank second only to lung cancer and to represent the most common malignancy in women. Neoadjuvant chemotherapy (NACT) provides substantial clinical advantages for locally advanced breast cancer: tumors initially deemed inoperable are rendered operable, the primary tumor stage is downstaged, and eligibility for breast-conserving surgery is increased among patients who are ineligible at presentation (2, 3). In addition, prognosis is estimated on the basis of response to NACT (4–6). The 2019 St Gallen consensus recommends NACT as a preferred initial treatment strategy for patients with stage II–III disease, particularly in HER2-positive (7) and triple-negative breast cancer (8).

Response to neoadjuvant therapy varies among patients with breast cancer. It is well established that achievement of pCR after NACT is linked with significantly better DFS and OS compared with non-pCR (9, 10). To date, beyond molecular subtype, no additional biomarker is consistently validated as a predictor of pCR. Therefore, identification of reliable and readily accessible predictors of response to NACT is required to improve prognostic assessment and to inform adjuvant treatment strategies.

Inflammation and nutritional condition are reported to be linked to cancer progression, treatment response, and survival (10, 11). The PNI is a composite marker calculated from serum albumin and peripheral blood lymphocyte count, and it is used to reflect systemic nutritional and inflammatory status (12). Emerging evidence suggests that a higher PNI is closely related to better clinical outcomes after NACT in BC (13). In a single-center cohort, 304 patients receiving NACT are included by Arici et al., and a higher pre-chemotherapy PNI is shown to predict improved OS and significantly longer DFS over 3 years of follow-up (14). In a multicenter retrospective study, Birsin et al. reported that an elevated pre-NACT PNI was associated with a higher pCR rate in 174 patients treated with NACT between 2010 and 2025 (15). Nevertheless, the clinical utility of PNI in NACT for BC remains to be further clarified.

A meta-analysis published by Prasetiyo et al. included nine studies published between 2014 and 2023 (16). It is concluded that a higher PNI is linked to improved OS in individuals with BC, whereas no meaningful association with DFS is found. However, whether PNI provides comparable prognostic information specifically in patients receiving NACT remains insufficiently defined. Therefore, the present meta-analysis is conducted by building on prior evidence and incorporating newly published studies to more comprehensively determine the clinical significance of pre-NACT PNI in BC.

## 2 Materials and methods

### 2.1 Search strategy

This meta-analysis adhered to the PRISMA guidelines (17). The study protocol was prospectively registered in PROSPERO (CRD420251268856). The literature retrieval plan was formulated by two investigators (MHS and ZQZ). PubMed, Embase, Web of Science, and the Cochrane Library were queried on November 1, 2025, to identify eligible studies, without language restrictions. In PubMed, search terms included those related to the prognostic nutritional index (e.g., “prognostic nutritional index,” “PNI”), breast cancer (e.g., “breast neoplasm,” “breast cancer,” “breast malignant neoplasm,” “mammary cancers”), and neoadjuvant treatment (e.g., “neoadjuvant therapy,” “neoadjuvant chemotherapy,” “neoadjuvant systemic therapy,” and “neoadjuvant radiation”). The full search procedures are provided in **Supplementary Table S1**.

### 2.2 Study selection

Eligibility criteria were defined according to the PICOS framework, which specifies the population, intervention, comparator, outcomes, and study design. Studies were considered eligible if (1): breast cancer was confirmed by pathological examination (2); neoadjuvant chemotherapy (NACT) was administered (3); the prognostic utility of the PNI on OS, DFS, and pCR was evaluated (4); HRs or ORs with 95% CIs, or sufficient data (e.g., P values) to derive effect estimates, were reported; and (5) participants were stratified into two groups according to PNI (e.g., low vs high, based on a defined cut-off). Studies were excluded if (1): hematologic malignancies were investigated (2); the publication was a review, commentary, conference abstract, case report, or letter (3); HRs for OS/DFS or ORs for pCR were not provided or could not be obtained; or (4) only individual PNI components were reported as continuous variables without group-based PNI stratification. When overlapping cohorts from the same center were identified, the study with the greatest enrollment was retained. Study selection was performed independently by two reviewers, and any disagreement was resolved through discussion.

### 2.3 Data extraction

Two investigators separately abstracted the data into a prespecified Excel form, and the extracted records were consolidated by a third reviewer. Disagreements were resolved through discussion. Data items recorded for every included investigation were as follows: first author, publication year, region, study design, sample size, patient age, study population, study period, treatment regimen, follow-up duration, TNM stage, PNI cut-off value, method used for cut-off determination, timing of PNI assessment, definition of pCR, and effect estimates for OS, DFS, and pCR (HRs or ORs with 95% CIs). When both univariable and multivariable hazard ratios were reported, multivariable estimates were preferentially extracted.

### 2.4 Quality assessment

Methodological quality was rated with the Newcastle–Ottawa Scale (NOS), which evaluates selection, comparability, and outcome domains. In the present analysis, the highest NOS score was 8 among the included studies (18, 19). Two reviewers carried out the quality appraisal separately, and any inconsistencies were addressed through discussion.

### 2.5 Statistical analysis

HRs for OS and DFS and ORs for pCR, together with 95% CIs, were extracted from each included study. Pooled effect estimates were synthesized using fixed- or random-effects models according to the degree of heterogeneity across studies. Heterogeneity was quantified with Cochran’s Q test and the Higgins I² statistic (20). Substantial heterogeneity was defined as I² > 50% or P < 0.05. A fixed effects approach was chosen when heterogeneity was not notable; otherwise, a random-effects approach was adopted. Potential drivers of heterogeneity were explored using subgroup analyses. Robustness was examined using leave-one-out sensitivity analyses. Publication bias was inspected by funnel plots and Egger’s test; when significant asymmetry was observed, trim-and-fill analyses were conducted to obtain bias-adjusted effects. All tests were two-sided, and P < 0.05 was considered statistically significant. Statistical analyses were performed using STATA (version 15.0) and Review Manager (version 5.4).

## 3 Results

### 3.1 Study selection and characteristics

**Figure 1:**
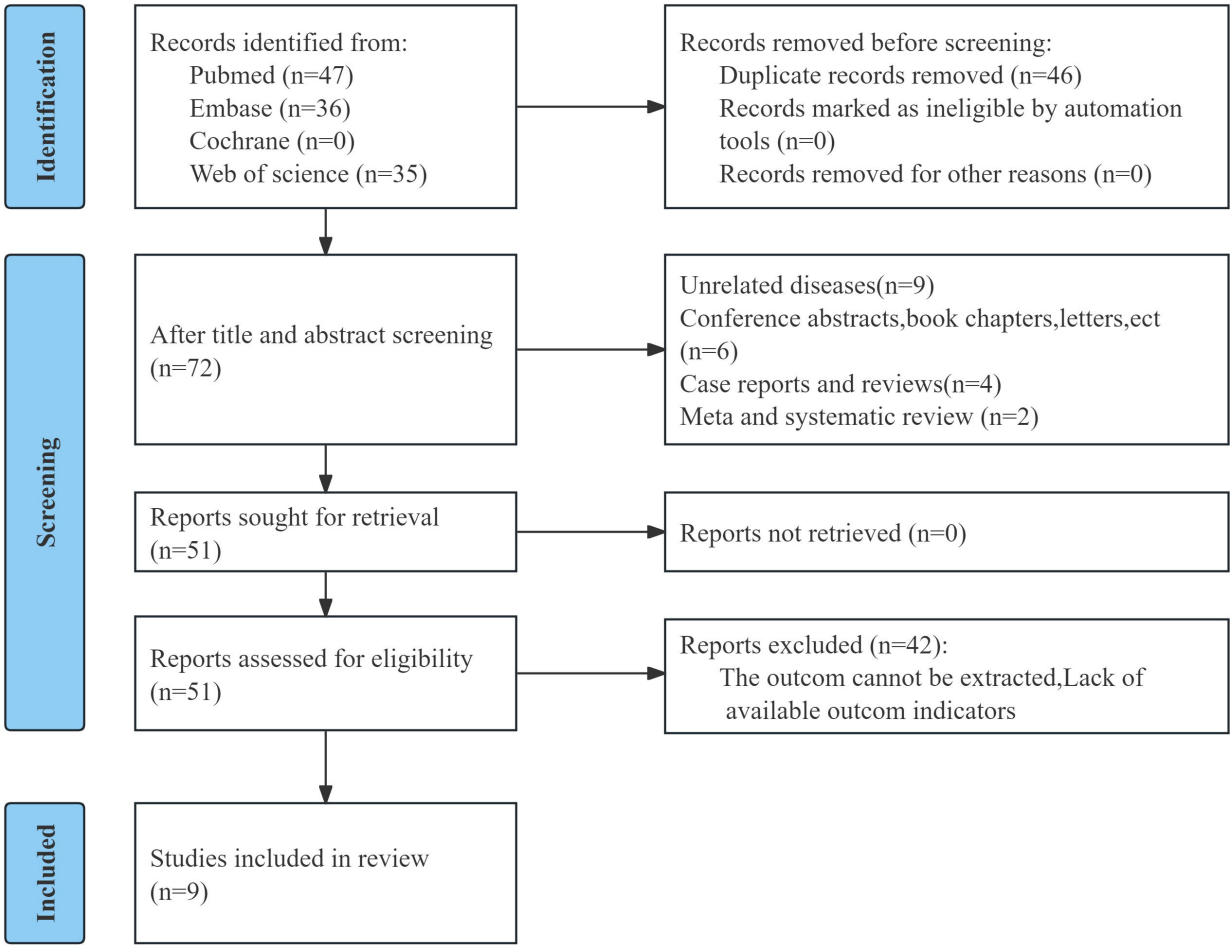
Flow chart of literature screening.

The initial search yielded 118 records, of which 72 remained after duplicate removal. After title and abstract screening, 21 records were excluded. Full texts of 51 articles were assessed, and 42 were removed. Ultimately, nine studies involving 3,718 patients were incorporated into the analysis (**Figure 1**) (13–15, 18, 19, 21–24). Sample sizes ranged from 110 to 1,170.

Across the nine included studies published between 2020 and 2025, 10 comparison groups were extracted. Four comparison groups were conducted in Asia (13, 21, 22, 24), four were conducted in non-Asian settings (14, 18, 23), and two were derived from multicenter cohorts (15, 19). All participants received NACT, and PNI was assessed before NACT in all comparison groups; however, the timing of PNI assessment was not reported in one study. All comparison groups were published in English and used retrospective designs, with study periods spanning 1998–2025. Median age ranged from 47 to 52 years, and PNI cut-off values ranged from 47 to 55. Most included studies determined the PNI cut-off using study-specific ROC curve analysis, indicating that the thresholds were largely data-driven rather than prespecified; in one study, the cohort median was used because ROC analysis did not identify a statistically significant threshold (**Table 1**). Associations between PNI and OS were reported in three studies, associations with DFS were evaluated in four studies, and associations with pCR were examined in six studies. Across the included studies evaluating pCR, the outcome referred to total pCR (tpCR), rather than breast-only or axillary-only pCR. Among the six studies evaluating pCR, two were conducted in subtype-restricted populations, specifically TNBC-only and HER2-positive cohorts. **Table 1** presents the main characteristics of the included studies.

**Table 1:** Basic characteristics of the included literature.

Author	Study period	Region	Study design	Population	Treatment method	Timing of detection	No. of patients	Median follow- up (months)	Mean/median age	TNM stage	PNI cut-off	Method for cut-off determination	Outcomes	Definition of pCR
Qu, F 2023 (19)	2012–2022	Multicenter	Retrospective cohort	BC	NACT and surgery	Pre-treatment	1170	NA	49.0	I-IV	53.0	ROC curve analysis with the maximum Youden index	pCR	tpCR
Wang, S 2025 (21)	2018–2023	China	Retrospective cohort	TNBC	NACT and surgery	Pre-treatment	431	NA	48.0	I-IV	53.6	ROC curve analysis	pCR	tpCR
Birsin, Z 2025 (15)	2010–2025	Multicenter	Retrospective cohort	HER2 positive BC	NACT and surgery	Pre-treatment	174	NA	51.0	II-III	55.0	Cohort median (ROC analysis non-significant)	pCR	tpCR
Buyuksimsek, M 2020 (18)	2006–2019	Turkey	Retrospective cohort	BC	NACT and surgery	Pre-treatment	110	NA	52.0	II-III	50.0	ROC curve analysis	pCR	tpCR
Oba, T 2020 (22)	2005–2016	Japan	Retrospective cohort	BC	NACT and surgery	NA	191	51	51.2	I-III	NA	ROC curve analysis	DFS	NA
Arici, M 2024 (14)	2015–2023	Turkey	Retrospective cohort	BC	NACT and surgery	Pre-treatment	304	38.5	50.0	II-III	54.1	ROC curve analysis	OS/DFS	NA
Chen, L 2021 (13)	1998–2016	China	Retrospective cohort	BC	NACT and surgery	Pre-treatment	477	55	47.0	I-III	51.0	ROC curve analysis	OS/DFS	NA
Yildirim, S 2024a (23)	2010–2022	Turkey	Retrospective cohort	BC	NACT and surgery	Pre-treatment	624	42	50.0	I-III	52.7	ROC curve analysis	OS/DFS	NA
Yildirim, S 2024b (23)	2010–2022	Turkey	Retrospective cohort	BC	NACT and surgery	Pre-treatment	624	42	50.0	I-III	54.0	ROC curve analysis	pCR	tpCR
Guo, X 2024 (24)	2010–2021	China	Retrospective cohort	BC	NACT and surgery	Pre-treatment	237	NA	50.0	II-III	47.0	ROC curve analysis	pCR	tpCR

### 3.2 Study quality

All included studies achieved NOS scores of ≥7, indicating generally robust study quality (**Supplementary Table S2**).

### 3.3 Meta-analysis results

#### 3.3.1 PNI and OS

**Figure 2:**
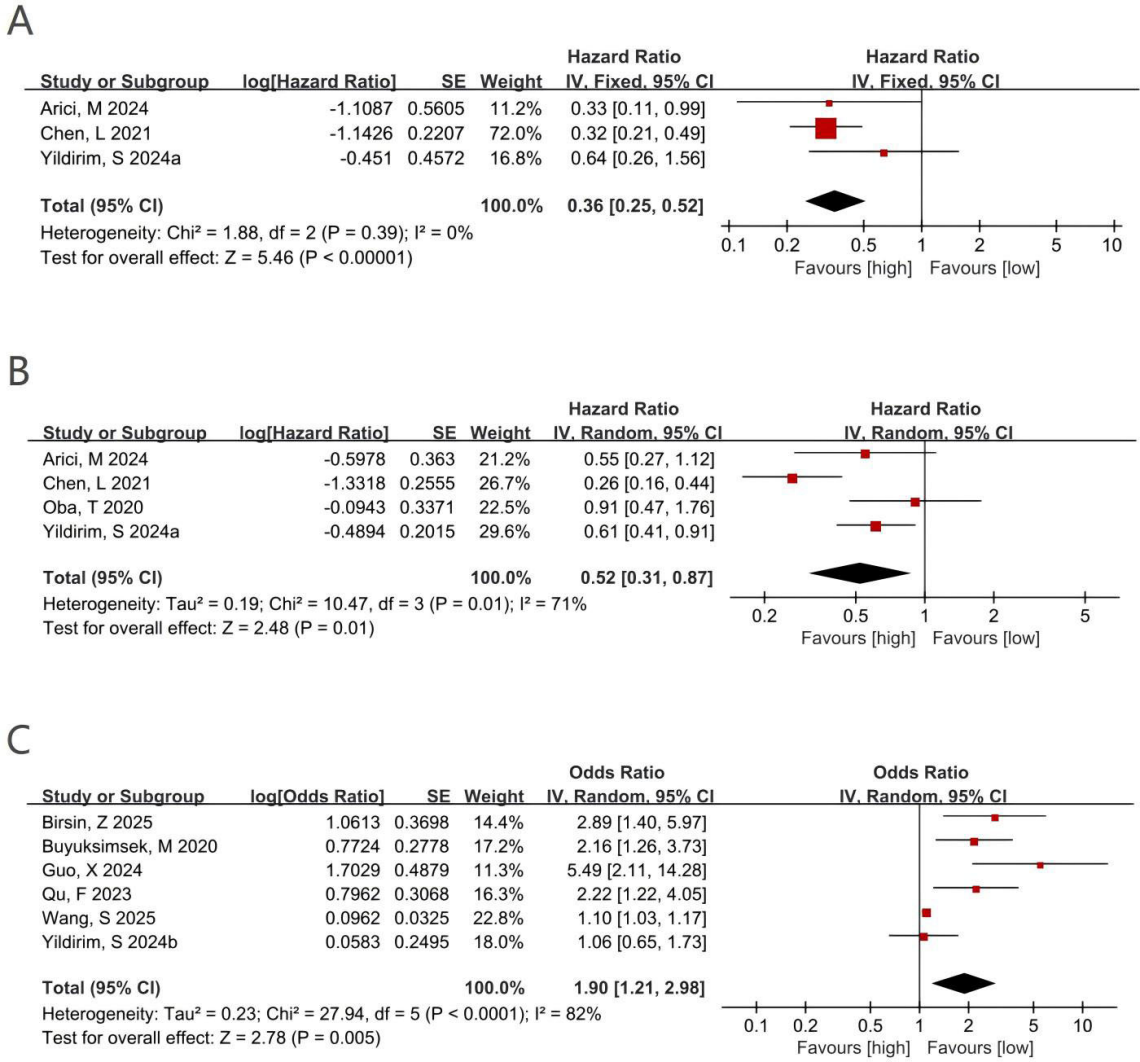
**(A)** Forest plots for the association between PNI and OS; **(B)** forest plots for the association between PNI and DFS; **(C)** forest plots for the association between PNI and pCR.

Data from three studies comprising 1,405 patients were available for the OS analysis. Compared with a low pre-treatment PNI, a high PNI was significantly associated with better OS (HR = 0.36; 95% CI 0.25–0.52; P < 0.0001) (**Figure 2A**). Negligible heterogeneity was detected across studies (I² = 0%; P = 0.39); therefore, a fixed-effect model was used. Because heterogeneity was absent, subgroup analyses were not performed.

#### 3.3.2 PNI and DFS

Four studies evaluated the link between pre-treatment PNI and DFS in individuals with BC receiving NACT. In the pooled analysis, higher PNI corresponded to a lower risk of DFS events (HR = 0.52; 95% CI 0.31–0.87; P = 0.01) (**Figure 2B**). Substantial between-study heterogeneity was detected (I² = 71%; P = 0.01); therefore, a random-effects model was chosen. To investigate possible contributors of heterogeneity, subgroup analyses were conducted by geographic region, sample size, PNI cut-off value, and age (**Table 2**). Overall, these subgroup analyses were generally consistent with the main finding; however, no significant association between PNI and DFS was found in studies conducted in Asia (P = 0.24). When stratified by cohort magnitude, the relationship between high PNI and improved DFS was observed in comparison groups with ≥400 participants (P = 0.03), whereas no significant relationship was observed in those with <400 participants (P = 0.19). Given the substantial heterogeneity and the lack of statistical significance in several subgroup analyses, the pooled association between pre-treatment PNI and DFS should be interpreted with caution.

**Table 2:** Pooled HRs/ORs for OS, DFS and pCR in subgroup analyses.

Subgroup	DFS				PCR			
	Study group	HR [95%CI]	*P* value	*I* ^2^	Study group	OR [95%CI]	*P* value	*I* ^2^
Total	4	0.52 [0.31, 0.87]	0.01	71%	6	1.90 [1.21, 2.98]	0.005	82%
Sample size								
<400	2	0.72 [0.44, 1.18]	0.19	3%	3	2.89 [1.78, 4.68]	<0.0001	28%
400	2	0.41 [0.18, 0.93]	0.03	85%	3	1.26 [0.89, 1.80]	0.2	61%
Mean/median Age.								
<50	1	0.26 [0.16, 0.44]	<0.00001	NA	2	1.46 [0.75, 2.87]	0.27	81%
≥50	3	0.65 [0.48, 0.89]	0.007	0%	4	2.27 [1.19, 4.30]	0.01	74%
Region								
Asia	2	0.48 [0.14, 1.62]	0.24	88%	2	2.28 [0.48, 10.95]	0.3	91%
Non-Asia	2	0.60 [0.42, 0.84]	0.003	0%	2	1.50 [0.74, 3.02]	0.26	73%
ulticenter	NA	NA	NA	NA	2	2.47 [1.56, 3.92]	0.0001	0%
PNI cut-off.								
<52	1	0.26 [0.16, 0.44]	<0.00001	NA	2	3.16 [1.29, 7.75]	0.01	64%
≥52	2	0.60 [0.42, 0.84]	0.003	0%	4	1.50 [0.98, 2.32]	0.06	75%

#### 3.3.3 PNI and pCR

Six studies comprising 2,746 patients were included to evaluate the relationship between pre-treatment PNI and pCR in individuals with BC receiving NACT. Substantial heterogeneity was observed (I² = 82%; P < 0.0001); therefore, a random-effects model was used. A high PNI was significantly associated with an increased probability of achieving pCR (OR = 1.90; 95% CI 1.21–2.98; P = 0.005) (**Figure 2C**). In subgroup analyses, no significant association was observed in comparisons using a cut-off value ≥52 (P = 0.06), in those with sample size ≥400 (P = 0.20), in cohorts with a median age <50 years (P = 0.27), or in studies conducted in Asia (P = 0.30) or non-Asian settings (P = 0.26). Significant associations were observed in the remaining subgroups. Between-study heterogeneity was suggested to be driven by differences in sample size, age, geographic region, and PNI cut-off values. Because two included studies were restricted to specific molecular subtypes, an additional sensitivity analysis excluding the TNBC-only and HER2-positive cohorts was performed. The association between high PNI and increased pCR remained statistically significant in the overall BC cohorts (OR = 2.10; 95% CI 1.18-3.76; P = 0.01)(**Supplementary Figure S1**), although substantial heterogeneity persisted (I² = 72%). These findings suggest that the pooled pCR result was not solely driven by the inclusion of subtype-restricted populations.

### 3.4 Sensitivity analysis

**Figure 3:**
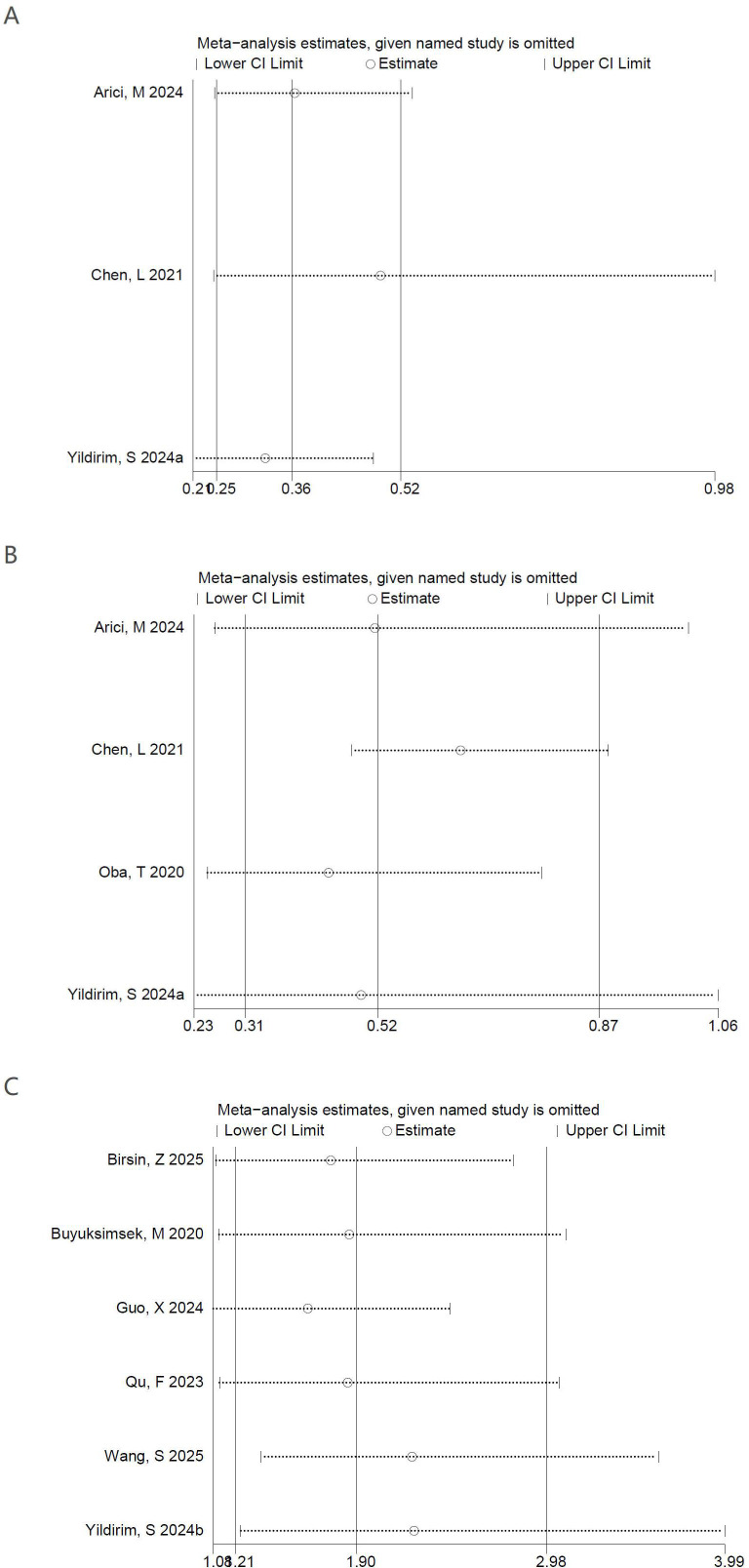
Sensitivity analysis of **(A)** OS, **(B)** DFS and **(C)** pCR.

Sensitivity analyses were carried out to verify the stability of the pooled estimates. The pooled effect sizes for OS (**Figure 3A**) and pCR (**Figure 3C**) remained statistically significant after sequential exclusion of individual studies, supporting the robustness of these findings. In contrast, for DFS (**Figure 3B**), the pooled estimate changed materially when the study by Arici et al. (14) (HR = 0.52; 95% CI 0.26–1.01) or Yildirim et al. (23) (HR = 0.50; 95% CI 0.23–1.06) was excluded. These findings indicate that, although the pooled association between PNI and DFS reached statistical significance in the primary analysis (P = 0.01), it was sensitive to the omission of individual studies and therefore should be interpreted with caution.

### 3.5 Publication bias

**Figure 4:**
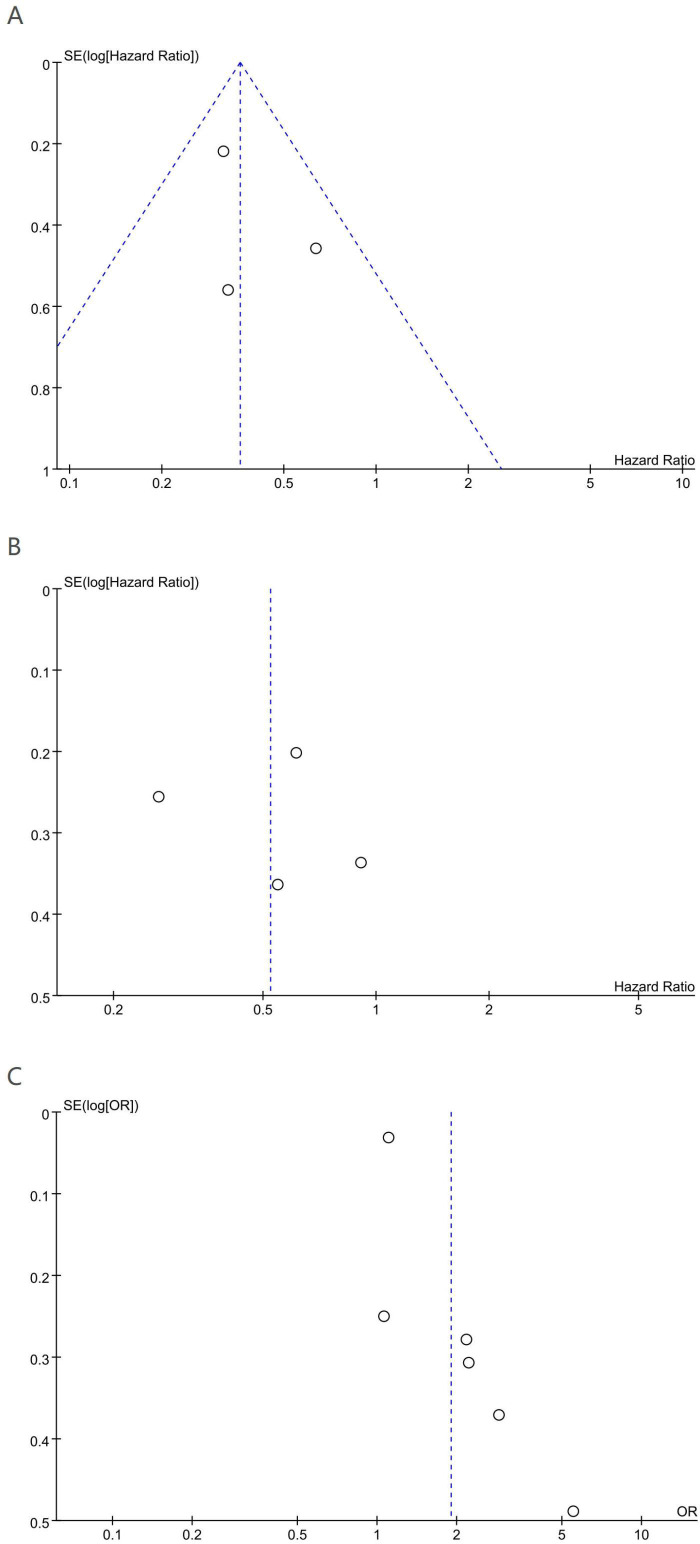
Funnel plot for the evaluation of publication bias for **(A)** OS, **(B)** DFS and **(C)** pCR.

Potential publication bias for OS, DFS, and pCR was appraised using funnel plots and Egger’s test. Visual inspection suggested no substantial asymmetry for OS (**Figure 4A**) or DFS (**Figure 4B**). Consistently, Egger’s test for OS was not significant (P = 0.60). For pCR, funnel plot asymmetry was observed (**Figure 4C**), and Egger’s test indicated statistical significance (P = 0.021), suggesting potential publication bias. Thus, the trim-and-fill approach was implemented to obtain a bias-adjusted effect. After adjustment, the association remained statistically significant (OR = 1.650; 95% CI 1.073–2.535; P = 0.022) (**Supplementary Figure S2**), indicating the stability of the pCR finding.

## 4 Discussion

In this meta-analysis, nine studies comprising more than 3,000 patients with BC were included to evaluate the prognostic relevance of the PNI measured before NACT. A high pre-NACT PNI was associated with longer OS and an increased likelihood of pCR, while a favorable association with DFS was also observed. However, the DFS finding should be interpreted with caution because substantial heterogeneity was present and the result showed limited stability in sensitivity analyses. To our knowledge, the prognostic role of pre-NACT PNI in BC has been systematically synthesized for the first time in the present work.

In recent years, the development of novel pre-NACT biomarkers for predicting survival outcomes has been increasingly prioritized in breast cancer research (25, 26). Prior meta-analyses reported that immune inflammatory indices (25, 27, 28), including the neutrophil-to-lymphocyte ratio (NLR) and lymphocyte-to-monocyte ratio (LMR), were correlated with outcomes in patients with BC treated with NACT (25, 29). Consistent with these findings, our meta-analysis suggested that a high PNI was associated with superior OS and a higher likelihood of pCR in the neoadjuvant setting. Although a statistically significant association was also observed for DFS, this result should be interpreted cautiously because of substantial heterogeneity and limited stability in sensitivity analyses. Consistently, Egger’s test suggested no indication of publication bias for OS or DFS, but potential publication bias for pCR. Notably, studies evaluating PNI as a prognostic marker in patients undergoing NACT began in 2020 (18), later than studies of NLR or LMR; therefore, the current evidence base remains limited. The prognostic utility of PNI within specific breast cancer subgroups remains to be clarified by additional well-designed studies.

The subgroup analyses further indicated heterogeneity in the prognostic performance of the PNI among individuals with BC undergoing NACT, and suggested several potential effect modifiers. In subgroup analyses based on age, a significant prognostic association was observed in patients aged ≥50 years, whereas no significant association was detected in those aged <50 years. This pattern may be attributable to immunosenescence and chronic low-grade inflammation in older adults, by which PNI is more likely to capture systemic physiologic reserve and treatment tolerance (30, 31). In region-stratified analyses, no significant association was observed in certain single-center regional subgroups. The absence of statistical significance may be explained by the small pool of included studies. It may also reflect population-level differences in genetic background, dietary patterns, health-care resources, and tumor biology. In addition, greater treatment standardization in multicenter cohorts may attenuate or obscure PNI-related effects. Therefore, additional international, multicenter studies are required, and standardized procedures for PNI assessment (including timing of blood sampling, albumin measurement, lymphocyte counting, and cut-off definition) are needed to determine whether prognostic performance is consistent across regions and clinical settings. With respect to cut-off selection, subgroup analyses suggested stronger prognostic discrimination when the PNI cut-off was <52 than when it was ≥52. These findings support the use of cut-offs below 52 in future risk models, or the adoption of context-specific thresholds calibrated to patient and disease characteristics (e.g., stage, treatment response, and age) to optimize prognostic accuracy.

Although substantial clinical evidence has supported low PNI as an adverse prognostic factor, the mechanisms linking PNI to outcomes in breast cancer remain incompletely understood. Biological plausibility is provided by the two components of PNI: serum albumin and peripheral lymphocyte count. Serum albumin is a marker of nutritional reserve and is involved in key physiological processes (32), including immune modulation and maintenance of oncotic pressure and fluid homeostasis (33, 34). In patients with cancer, reduced hepatic synthetic function, inadequate intake, and metabolic dysregulation are associated with hypoalbuminemia (35, 36), which is linked to impaired host immunity and unfavorable oncologic outcomes (37, 38). Lymphocytes are central mediators of antitumor immunity and contribute to the elimination of malignant cells and the restriction of tumor dissemination (39). Tumors frequently induce immune-evasion pathways that suppress lymphocyte activity, thereby facilitating progression and metastasis (40). Tumor-infiltrating lymphocytes are also reported to be strongly linked to prognosis and response to chemotherapy (41–43). Collectively, a high PNI—reflecting higher albumin levels and increased lymphocyte counts—is plausibly linked with better survival in patients with BC.

Several limitations of the current analysis merit careful consideration. First, considerable between-study heterogeneity was noted, particularly in the analyses of PNI with DFS and pCR, which may limit the stability and generalizability of these findings. The DFS result was sensitive to leave-one-out analyses, indicating limited robustness. In addition, because pCR is strongly influenced by molecular subtype in breast cancer, the inclusion of TNBC-only and HER2-positive cohorts may have introduced additional clinical heterogeneity. Although the association between high PNI and increased pCR remained significant after exclusion of these subtype-restricted cohorts (OR = 2.10; 95% CI 1.18-3.76; P = 0.01), substantial heterogeneity persisted. Residual confounding related to interstudy differences in patient characteristics, molecular subtype distribution, and neoadjuvant treatment regimens therefore cannot be fully excluded. Second, all included studies used retrospective designs, and selection and measurement biases may therefore be introduced. In addition, key clinical variables—such as the type of operation and postoperative adjuvant treatment—were often insufficiently reported, which constrains interpretation and clinical translation. Third, because PNI is computed by serum albumin and peripheral lymphocyte count, it is susceptible to distortion by intercurrent infection, comorbid conditions, concomitant medications, and laboratory assay variability; these factors were frequently not recorded or adequately adjusted for in the primary studies. Fourth, PNI cut-off values were not standardized across studies, and the methods used to determine these thresholds also varied. In most included studies, the cut-offs were derived using study-specific ROC curve analysis, suggesting that they were largely data-driven rather than prespecified, whereas one study used the cohort median after ROC analysis failed to identify a statistically significant threshold. This lack of standardization reduces comparability and may have contributed to between-study heterogeneity. Finally, although the pCR association remains significant after trim-and-fill adjustment, Egger’s test suggests potential publication bias, indicating that studies with null results may be underreported or underrepresented.

Prospective, multicenter studies with large sample sizes are required to standardize the timing of PNI assessment, calculation procedures, and cut-off definitions, and to ensure systematic collection of treatment details, complications, and concomitant medication use, so that the prognostic value of PNI in the neoadjuvant setting for BC is more rigorously validated. In addition, the clinical relevance of strategies designed to raise PNI—such as nutritional support and immunomodulatory interventions—should be investigated to determine whether optimization of PNI translates into improved patient outcomes.

## 5 Conclusion

In summary, a high PNI measured before NACT was significantly associated with superior OS and a higher likelihood of pCR in patients with BC. A favorable association with DFS was also observed, although this finding should be interpreted cautiously because of substantial heterogeneity and limited stability in sensitivity analyses. These results support the potential utility of PNI as a simple and practical prognostic biomarker in the neoadjuvant setting. Further prospective, multicenter studies are needed to validate its prognostic value in broader populations and to determine whether its predictive performance can be improved by integration with established clinical and molecular markers for risk stratification and individualized treatment.

## Data Availability

The original contributions presented in the study are included in the article/**Supplementary Material**. Further inquiries can be directed to the corresponding authors.
