# Supplementary figures and images for: Prognostic value of prognostic nutritional index in breast cancer patients receiving neoadjuvant therapy: a systematic review and meta-analysis
Source: Front Oncol. 2026 Apr 27;16:1775749. doi: 10.3389/fonc.2026.1775749 (PMC13158091; doi:10.3389/fonc.2026.1775749)

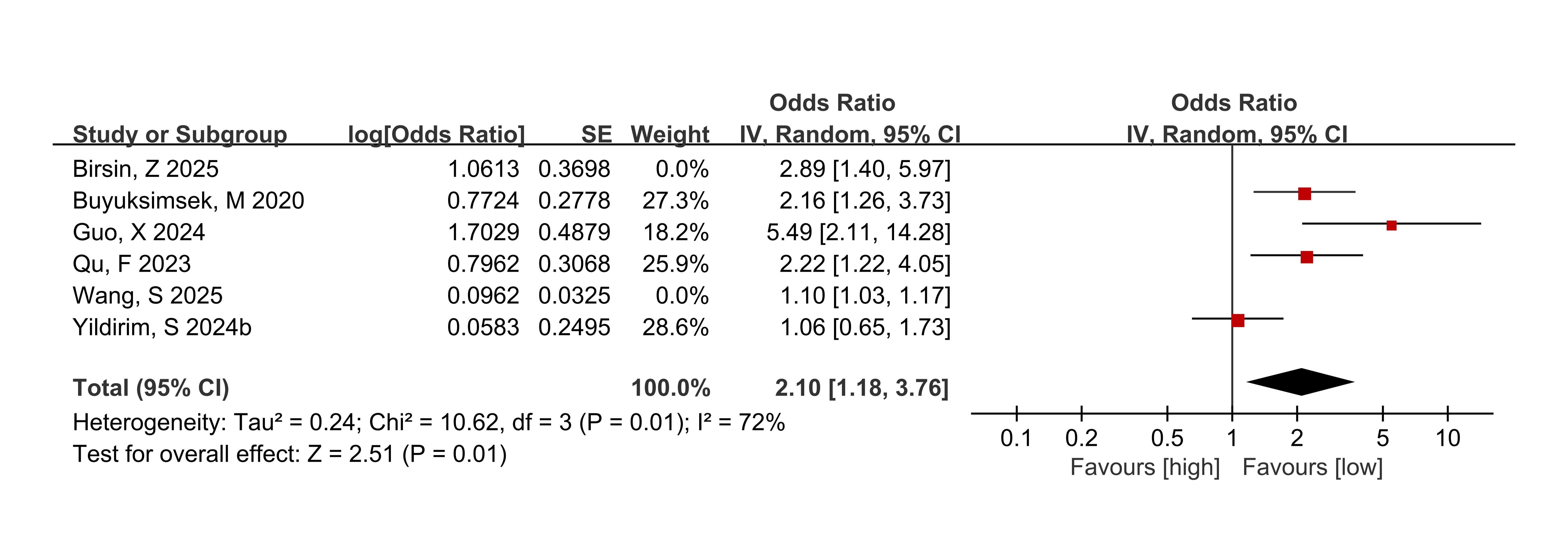

Supplement: Supplementary file 1 [file Image1.jpeg]

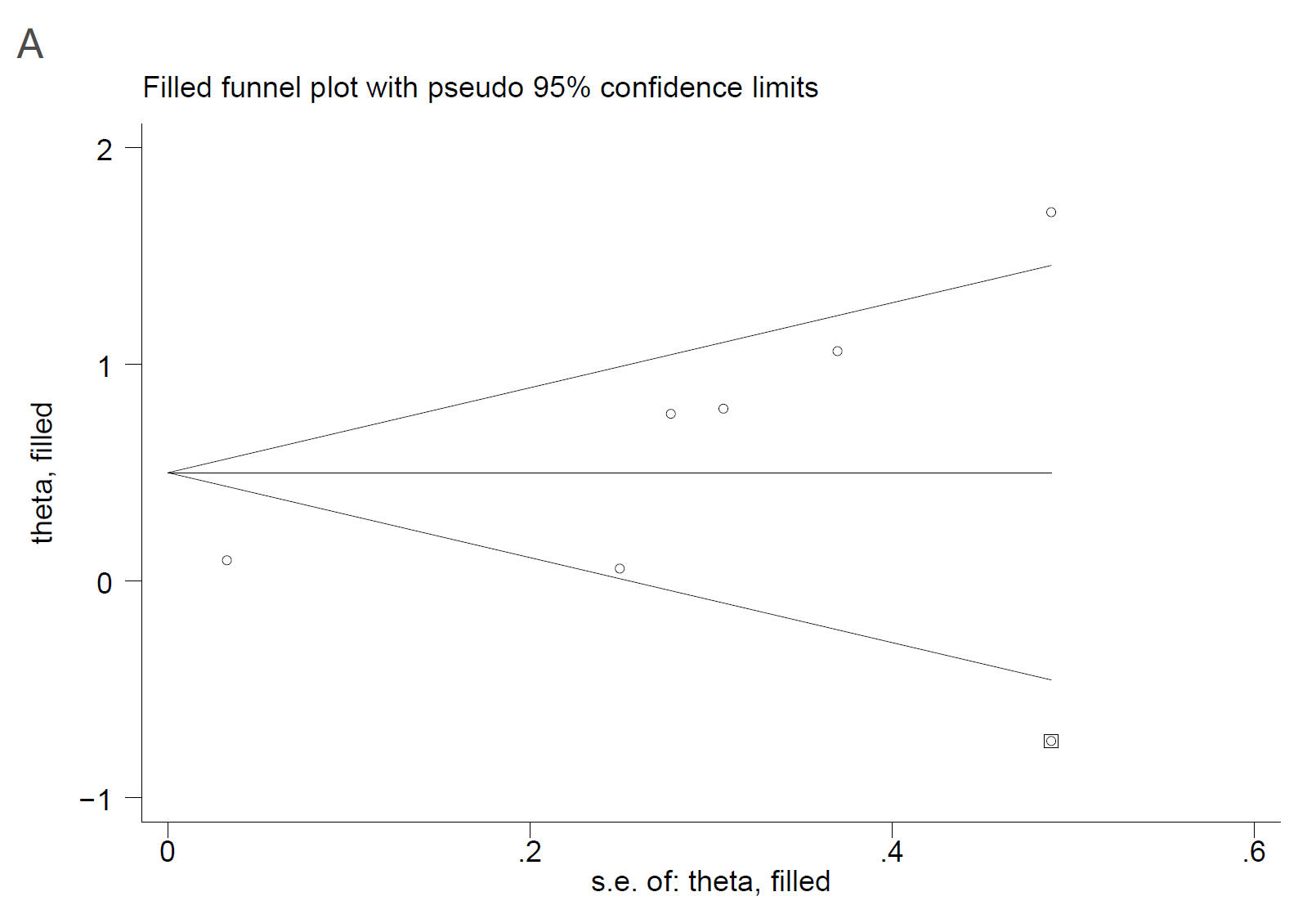

Supplement: Supplementary file 2 [file Image2.jpeg]
