# Supplementary material for: Prognostic value of prognostic nutritional index in breast cancer patients receiving neoadjuvant therapy: a systematic review and meta-analysis
Source: Front Oncol. 2026 Apr 27;16:1775749. doi: 10.3389/fonc.2026.1775749 (PMC13158091; doi:10.3389/fonc.2026.1775749)
Supplement: Supplementary file 4 [file Table2.docx]

| Supplementary Table S2. Quality evaluation of the eligible studies with Newcastle–Ottawa scale. | | | | | | | | | |
| --- | --- | --- | --- | --- | --- | --- | --- | --- | --- |
| Study | Selection | | | | Comparability | | Outcome | | |
|  | Representative-ness | Selection of  non-exposed | Ascertainment  of exposure | Outcome not present at start | Comparability on most important factors | Comparability on other risk factors | Assessment of outcome | Long enough follow-up (median≥1 year) | Adequacy  (completeness) of follow-up |
| Qu, F et al. | * | * | * | * | * | - | * | * | * |
| Wang, S et al. | * | * | * | * | - | - | * | * | * |
| Birsin, Z et al. | * | * | * | * | - | - | * | * | * |
| Buyuksimsek, M et al. | * | * | * | * | - | * | * | * | * |
| Oba, T et al. | * | * | * | * | - | - | * | * | * |
| Arici, M et al. | * | * | * | * | - | - | * | * | * |
| Chen, L et al. | * | * | * | * | - | - | * | * | * |
| Yildirim, S et al. | * | * | * | * | - | - | * | * | * |
| Guo, X et al. | * | * | * | * | - | - | * | * | * |
| *indicates criterion met; - indicates significant of criterion not met. | | | | | | | | | |
